# Retraction: Prmt1-mediated histone H4R3me2a methylation regulates the proliferation, migration and invasion of laryngeal cancer cells by affecting the expression level of NCOA5

**DOI:** 10.3389/fonc.2026.1960998

**Published:** 2026-08-17

**Authors:** Frontiers Editorial Office

The journal retracts the article cited above.

Following publication, concerns were raised regarding the integrity of the images in the published figures. Image duplication concerns were identified in Figure 1C and Figure 2C.

The authors failed to provide a satisfactory explanation during the investigation, which was conducted in accordance with Frontiers’ policies. As a result, the data and conclusions of the article have been deemed unreliable, and the article has been retracted.

This retraction was approved by Chief Executive Editor of Frontiers. The authors agree to this retraction.

Frontiers would like to thank Sholto David for contacting the journal regarding the published article.

